# A Comprehensive Review of Techniques for Processing and Analyzing Fetal Heart Rate Signals

**DOI:** 10.3390/s21186136

**Published:** 2021-09-13

**Authors:** Alfonso Maria Ponsiglione, Carlo Cosentino, Giuseppe Cesarelli, Francesco Amato, Maria Romano

**Affiliations:** 1 Department of Electrical Engineering and Information Technology (DIETI), University of Naples Federico II, Via Claudio 21, 80125 Naples, Italy; alfonsomaria.ponsiglione@unina.it (A.M.P.); framato@unina.it (F.A.); 2 Department of Experimental and Clinical Medicine ‘Gaetano Salvatore’, University Magna Graecia of Catanzaro, Viale Tommaso Campanella 185, 88100 Catanzaro, Italy; carlo.cosentino@unicz.it; 3 Department of Chemical, Materials and Production Engineering (DICMaPI), University of Naples Federico II, Piazzale Tecchio 80, 80125 Naples, Italy; giuseppe.cesarelli@unina.it

**Keywords:** fetal heart rate, fetal heart rate variability, biomedical signal processing and analysis, linear FHRV indices, nonlinear FHRV indices, artificial neural networks

## Abstract

The availability of standardized guidelines regarding the use of electronic fetal monitoring (EFM) in clinical practice has not effectively helped to solve the main drawbacks of fetal heart rate (FHR) surveillance methodology, which still presents inter- and intra-observer variability as well as uncertainty in the classification of unreassuring or risky FHR recordings. Given the clinical relevance of the interpretation of FHR traces as well as the role of FHR as a marker of fetal wellbeing autonomous nervous system development, many different approaches for computerized processing and analysis of FHR patterns have been proposed in the literature. The objective of this review is to describe the techniques, methodologies, and algorithms proposed in this field so far, reporting their main achievements and discussing the value they brought to the scientific and clinical community. The review explores the following two main approaches to the processing and analysis of FHR signals: traditional (or linear) methodologies, namely, time and frequency domain analysis, and less conventional (or nonlinear) techniques. In this scenario, the emerging role and the opportunities offered by Artificial Intelligence tools, representing the future direction of EFM, are also discussed with a specific focus on the use of Artificial Neural Networks, whose application to the analysis of accelerations in FHR signals is also examined in a case study conducted by the authors.

## 1. Introduction

Electronic fetal monitoring (EFM), commonly known as cardiotocography (CTG), was developed in the 1960s with the aim of assessing a fetus’s health before and/or during labor. The fetal status is assessed through the monitoring of fetal heart rate (FHR) and uterine contractions simultaneously. Many research works can be found around this issue; however, this topic is still important and interesting since biomedical research aims to continuously improve diagnostic techniques. Moreover, despite its widespread use, there is not yet a final shared consensus about neither the best recording procedure (even if photoplethysmography seems to be very promising) [1] nor the best signal processing technique [2].

FHR evaluation is crucial in clinical environments because it does not only reflect the behavior of the cardiovascular system but can also provide indirect information about the status of the Autonomous Nervous System (ANS), which controls the circadian rhythms, and indications about the neural development of the fetus [3,4,5]. Moreover, during labor, it can help to identify hypoxia, which can lead to newborn acidemia. In particular, the variability of the FHR (FHRV) has an intimate relationship with the development and functioning of the ANS since a large proportion of FHRV can be explained on the basis of centrally mediated fluctuations in the parasympathetic and sympathetic tone [6].

Many different techniques can be adopted to measure the FHR [1], from fetal Electrocardiography (fECG) to fetal Phonocardiography (fPCG) and fetal Magnetocardiography (fMCG). Among them, CTG is still the most widespread, and it is considered the gold standard at the end of the pregnancy; furthermore, it is worth remembering that, in some countries, it represents a legal report. CTG is a non-invasive and simple tool that allows the simultaneous recording of both FHR and uterine contractions (UC) using Doppler ultrasound and pressure sensors. However, a common problem in the CTG and, more generally, in the EFM, is the poor inter- and intra-observer reliability as well as the high rates of false positives due to the visual interpretation of FHR recordings, which can lead to unnecessary operative deliveries and cesarean sections [1,3]. Therefore, many semiautomatic or automatic algorithms have been developed for the analysis of CTG traces in order to exploit all the potential hidden in the FHR and UC signals and extract reliable and useful information to improve the diagnostic efficacy [7,8,9,10,11].

Concerning FHR and FHRV assessment, many approaches are available. The techniques in the time and frequency domain are the most ‘traditional’ ones. However, very soon, it was understood that these techniques were not sufficient to highlight nonlinear phenomena underlying the FHRV physiology. Thus, nonlinear techniques began to be explored in the 1990s. Very briefly, it is possible to state that firstly, chaotic and fractal analyses were employed [12,13,14]. Then, since the 2000s, a great effort has been devoted to the study of entropy by proposing different methodologies and measures [15,16,17]. Finally, artificial neural network (ANN) and support vector machines (SVM) have been adopted, mainly for signals classification, i.e., for distinguishing and grouping FHR traces mainly according to the health status of the fetus (e.g., in order to distinguish between healthy and pathological signals) [18,19,20,21]. Simultaneously, other domain transformations, such as wavelet and Hilbert [3,22,23] and other techniques, such as Poincaré maps, Symbolic Dynamics (SD), Autoregressive models (AR), and others, have been applied for FHR analysis [24,25,26,27,28,29,30].

In spite of this wide availability of techniques and approaches for FHRV assessment, only weak predictive indications about fetal hypoxia and neonatal injuries can be achieved through the algorithms and methodologies developed so far [17]. In addition, while there are multiple, and also recent, examples of literature studies that review and discuss the available methodologies and metrics used both in research and in clinical practice to analyze the heart rate variability (HRV) of adult subjects [31,32,33,34], the field of fetal heart monitoring, despite a few examples of works focused on the neonatal and perinatal medicine [35,36], still lacks a comprehensive overview of the techniques employed in the analysis of FHRV.

In this work, a review of the most widespread signal processing techniques proposed in the literature to improve the analysis of FHRV, and hence the diagnosis of healthy fetal status, is carried out, regardless of the recording technique. An overview of the classical methods employed for FHR analysis is provided, then the so-called nonlinear techniques are analyzed. A summary of the most representative studies and the main results achieved by both traditional and nonlinear techniques is also given. Moreover, advantages, drawbacks, and predictive power of the reviewed methodologies are reported, and perspectives in the use of innovative alternative approaches rising from the field of Artificial Intelligence (AI), with particular regard to machine learning techniques, are discussed.

Finally, for some methodologies, new results obtained by the authors by means of the computerized analysis of CTG signals included in a database recorded in a clinical environment, and already employed for previous publications [37,38,39], will be shown.

## 2. Methods

### 2.1. Eligibility Criteria and Information Sources

The inclusion criteria are:

- Date of publication not older than 1990;
- Date of publication not newer than 2020;
- Kind of FHR recording and processing technique explicitly cited;
- Details about obtained results clearly reported.

To explore the main signal processing techniques currently employed, we have first consulted the Web of Science database (by Clarivate Analytics) as the main source of information. Then, we integrated the search by including additional references from multiple sources (Scopus, PubMed, Google Scholar).

### 2.2. Search Strategy

The search was performed by using the following main keywords: FHR, FHR variability, FHRV, nonlinear FHRV indices, linear FHRV indices.

The review focused on the following techniques for FHRV analysis, which emerged during the search and were ordered and examined according to their relevance and frequency of occurrence:

- Time domain analysis;
- Frequency domain analysis;
- Fourier Transform, Fast Fourier Transform (FFT), Short Time Fourier Transform (STFT);
- Autoregressive models;
- Wavelet transform (WT);
- Entropy indices;
- Symbolic Dynamics;
- Fractal analysis;
- Detrended Fluctuation Analysis (DFA);
- Poincaré maps;
- Hilbert and Hilbert–Huang transform;
- Complexity of Lempel Ziv (LZ);
- Markov models;
- Lyapunov exponent;
- Lomb method;
- Matching Pursuit (MP).

After describing the literature review, we put the lights on a further methodology for FHR analysis, which has received stronger attention mostly in recent years, namely, the Artificial Neural Networks (ANNs). Although they were not part of the basic search criteria, they were included as a cutting-edge methodology in this field.

### 2.3. Selection Process

**Figure 1:**
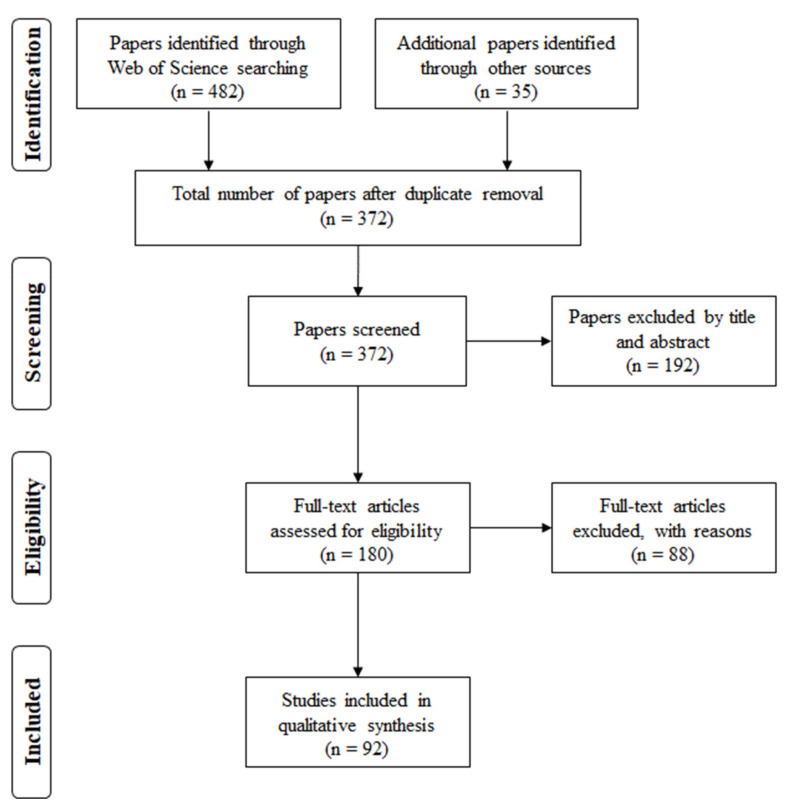
Diagram describing the study selection process.

Figure 1 shows a schematic representation of the selection process.

Among the 372 papers considered, 192 were excluded during the screening phase due to accessibility reasons or because they were out of the review scope. Then, 180 full-text papers were assessed for eligibility, and among them, 88 were excluded because they were not informative enough for the purpose of this review, or because they were too similar to other works by the same authors, or because they represented techniques not sufficiently described, or again because they concerned too-particular cases or focus on animal models rather than human fetuses. Finally, 92 studies were included in the qualitative synthesis.

### 2.4. Characteristics of the Screened Studies

**Figure 2:**
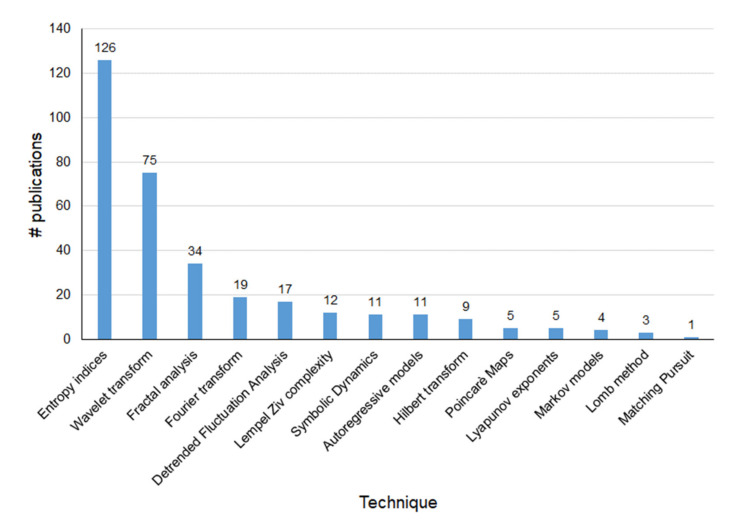
Number of publications identified through the Web of Science database grouped on the basis of the technique used for FHR processing and analysis (for better readability, only the time interval from 2000 to 2020 is shown).

**Figure 3:**
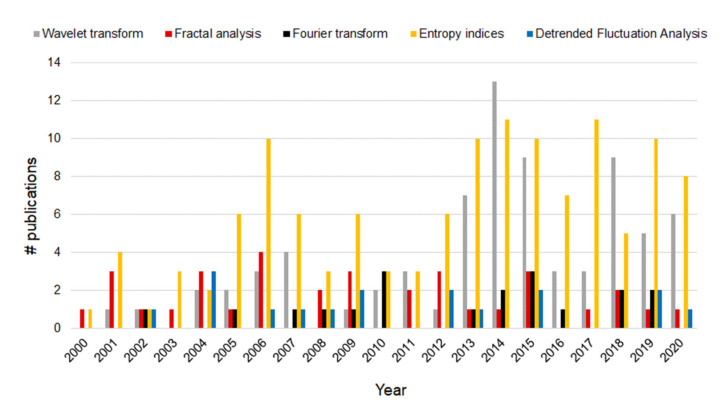
Annual distribution of the number of publications identified through the Web of Science database (for better readability, only the five most employed techniques in the period 2000–2020 are shown).

In Figure 2 and Figure 3, histograms of the most employed FHR processing and analysis techniques according to Web of Science database are shown. Even if the search considered papers since 1990, for the sake of readability, in both the figures, we report only the time interval 2000–2020.

It is worth performing some observations. Figure 3 shows that, according to the records found in the Web of Science database, the Lomb method is among the less used together with MP, Markov models, and Lyapunov exponents; indeed, despite its suitability for the processing of uneven series, the Lomb method is quite complex and time-consuming. Besides, the Poincaré method, which is enumerated among the most widespread techniques for the analysis of heart rate variations in adult subjects, is little applied to the analysis of FHR. Finally, Fourier analysis, due to its shared and recognized importance and simplicity, is considered apart from the more general frequency domain analysis. Instead, Figure 2 puts in evidence that the number of publications using the Fourier analysis keeps almost constant over the years. As far as fractal analysis and DFA, they have not been uniformly employed within the considered timespan, and the number of publications has not increased over time. On the contrary, the use of WT and entropy indices has seen a considerable rise, especially in recent years.

### 2.5. Characteristics of the Included Studies

The following Table 1 shows a qualitative synthesis with the characteristics of the most representative studies included in this review grouped according to the adopted approach: (i) time domain measurements; (ii) frequency domain analysis; (iii) nonlinear methods. A fourth category named ‘other methods’ has also been used to include less-widespread or less-common techniques for FHRV analysis. Finally, as previously mentioned, the last category is focused on the use of ANNs in the field of FHR monitoring. It is worth noting that, despite being less employed in the FHRV domain, the Poincaré maps have been included among the main nonlinear methods for their historically recognized value as powerful indices of HRV in adults.

Based on the analysis carried out, it is possible to state that, regardless of the methods exploited in isolated cases, the most employed signal processing techniques for FHR signals are: Fourier analysis, through different implementations; AR models; wavelet transform, continuous or discrete; sample entropy; and other ‘nonlinear’ techniques, such as Poincaré plots, fractal analysis, and others. Generally, since most of the methodologies analyzed have been developed in the context of studies on adult subjects, the starting point to conduct the HR analysis is the construction of the so-called ‘tachogram’, i.e., the sequence of durations of the inter-beat intervals (RR), meant as the distance between two following *R* peaks on the electrocardiogram [105], whereas when the analysis is carried out on fetuses and the signals are provided by cardiotocographs, the FHR is directly available.

## 3. State of the Art

In the following, we will provide the key information and relevant results about the use of the above-mentioned methodologies in the literature.

### 3.1. Time Domain Indices

The main processes, developed in the time domain, try to implement the FHR analysis in a way similar to that used by a physician who follows specific guidelines (for example, FIGO guidelines or others [106,107,108,109]) and medical terminologies. In particular, FHR baseline, accelerations, and decelerations are searched. To this aim, a variety of methods have been proposed using specialized algorithms [7,8,9,10,40]. Another approach concentrates on the derivation of indices from the FHR signal that is able to characterize the condition of the fetus, such as statistical metrics, able to quantify different facets of the FHRV, which are promoted by different autonomic sources. The most used ones are the mean and standard deviation of the FHR signal. Moreover, other indices are also used: the short-term variability (STV), which quantifies FHR variability over a very short time scale, usually on a beat-to-beat basis; the long-term irregularity (LTI), which is usually computed on a 3-min segment of RR sequence; and the Interval Index (II), which is, together with the LTI, is an index to quantify the long-term variability in the FHR [25].

According to Cesarelli et al. [41], there is no agreement on the formula to quantify the STV. Therefore, here we propose three formulas available in the literature. The first one is proposed by Organ et al. [42]:

(1) $$\overset{n}{\underset{i=1}{\sum }}\frac{\left|F\left(i+1\right)-F\left(i\right)\right|}{n}$$

where *n* is number of beats in 30 s, *F(i)* is the instantaneous FHR expressed in beats per minute (bpm) and obtained as 60,000/*T(i)*, with *T(i)* representing the instantaneous inter-beat intervals expressed in milliseconds.

The second one is proposed by Redman [43]:

(2) $$\frac{1}{15}\overset{15}{\underset{m=1}{\sum }}\left|R\left(m+1\right)-R\left(m\right)\right|$$

with *R(m)* calculated as:

(3) $$R\left(m\right)=\overset{r}{\underset{i=1}{\sum }}\frac{\left|T\left(i+1\right)-T\left(i\right)\right|}{r}$$

where *r* is the number of RR intervals in 3.75 s. It is worth highlighting the meaning of the factor 1/15 preceding the summation, which reflects the number of subintervals in 60 s as reported in [41].

The third one is proposed by Cesarelli et al. [41], and it is computed as the standard deviation of the FHR:

(4) $${\left[\frac{1}{n-1}\overset{n}{\underset{i=1}{\sum }}{\left(F\left(i\right)-\bar{F}\right)}^{2}\right]}^{1/2}$$

where *n* is the number of beats in 60 s and *F* is the mean of the FHR signal.

As far as the LTI and the II indices are considered, the following formulas in Equations (5) and (6) are proposed by Esposito et al. [44]:

(5) $$\mathrm{LTI}=m_{24}\left(j\right)=\sqrt{T_{24}^{2}\left(j\right)+T_{24}^{2}\left(j+1\right)}$$

defined as the interquartile range (1/4; 3/4) of the distribution of the modal *m*_24_*(j)* computed on a 3 min segment of inter-beat sequence *T*_24_*(i)* (with *i* = 1, …, 72).

(6) $$\mathrm{II}=\frac{\mathrm{std}\left[T_{24}\left(i+1\right)-T_{24}\left(i\right)\right]}{\mathrm{STV}}$$

calculated as the coefficient of variation between the differences of all FHR values in 1 min of inter-beat sequence *T*_24_*(i)* in ms (with *i* = 1, …, 23), taken each 2.5 s.

It is worth noting that usually, the STV is not directly used as a classifier but rather as a fetal well-being parameter and, therefore, as a reference measurement for the assessment of other indices. However, Pardey et al. [9] showed that STV correlates well with the development of metabolic acidemia and intrauterine death and could be used as a useful indicator in FHR monitoring. In addition, they state that a reduction in STV is superior to decelerations as a predictor of outcome and more comprehensive than umbilical artery Doppler velocimetry. In the same work, they also report that, in the absence of a sinusoidal rhythm, STV and long-term variability (LTV) are strongly correlated, while a low-frequency sinusoidal rhythm increases the LTV more than the STV.

While STV appears to be a valuable predictive antenatal tool, its effectiveness is influenced by frequencies oscillations and, in some cases, cannot distinguish the low FHR variations of healthy fetuses during quiet sleep from the low variations of compromised fetuses, as reported in the study of Cattani et al. [22]. On the other hand, long-term variability indices can be harder to quantify numerically.

Finally, as emerged from a recent systematic review on the role of STV in fetal growth restriction [45], there is not enough evidence supporting an association between STV and short- or long-term fetal and neonatal outcome, and more studies comparing STV and visual CTG examinations are needed before STV can be effectively used as a prognostic index in the clinical practice.

### 3.2. Frequency Domain Analysis

The frequency domain analysis quantifies the extent of contribution of each frequency component to the overall heart rate fluctuation. Basically, frequency domain analysis involves the calculation of the spectral energy content of each frequency component through a power spectral analysis (PSA).

PSA is exploited as a predictive tool in adult cardiac functions, and it has also been used to analyze the FHRV. Compared to time domain measurements, PSA is not only an index of the frequency distribution but, since power is a function of amplitude, it is also a measure of the degree of heart rate variations [6].

Similar to the adults’ HRV, even if with different ranges, in the FHRV analysis, it is usual to consider three frequency bands: Very Low Frequency (VLF: 0–0.05 Hz), Low Frequency (LF: 0.05–0.2 Hz), associated with the sympathetic control and vasomotor activity; and High Frequency (HF: 0.2–1 Hz), driven by respiration and mediated by vagal activity. Despite the availability of the above-mentioned band definitions, no agreements still exist in the literature about the precise range of values for each spectrum band, as already outlined in a previous review [5]. In addition, in some works [17,47,48], a different spectral band is sometimes considered, associated with fetal movements and the mechanical influences of the maternal breathing, the Movement Frequency (MF: 0.15–0.5 Hz).

The ratio between LF and HF, or the sum HF + MF, when the latter is used, represents an index of the balance between cardiac physiological control components (by the two branches of ANS) and fetus activity level (sympatho–vagal balance).

The most used methods to estimate the power spectral density (PSD), obtained by calculating powers and peak frequencies for the different frequency bands, can be divided into [46]: non-parametric techniques, e.g., Fourier-transform-based, and parametric techniques, e.g., Autoregressive (AR) models.

A brief description of the mentioned methods is given in the following paragraphs.

#### 3.2.1. Fast Fourier Transform and Short-Time Fourier Transform

The Fourier analysis is the most important and widespread methodology in the analysis of FHRV. Standard Fourier analysis basically consists of a decomposition of the signal into sinusoids.

Since the FHR signal is inherently a sampled variable, the discrete Fourier transform (DFT) allows the expression of the original FHR sequence in the discrete frequency domain. A widely used algorithm to compute the DFT is the FFT. The FFT works well on a short time scale as the stationarity of the FHRV signal is an essential requirement (often a signal segment of 32 s is chosen [39,50]). However, in the presence of non-stationary RR sequences, the hypotheses underlying the spectral analysis methods are no longer valid. Therefore, among various techniques for time-frequency analysis in non-stationary conditions developed so far, the STFT represents a variation of the Fourier transform particularly suitable for dealing with non-stationarity. The STFT breaks the signal into specific periods of constant length and applies the Fourier transform individually on each of these parts of the signal. The STFT is given as:

(7) $$X\left(\tau ,\omega \right)=\mathrm{STFT}\left\{x\left(t\right)\right\}=\int_{-\infty }^{+\infty }x\left(t\right)w\left(t-\tau \right)e^{-jt\omega }dt$$

In the above formula, the signal to be transformed *x(t)* is multiplied by a windowing function *w(t)* (usually a Hanning or Gaussian function), and the resulting signal is locally transformed as the windowing function shifts along the time axis. A two-dimensional function *X(τ, ω)* is then obtained, which is the Fourier Transform of *x(t)w(t − τ)* and represents phase and magnitude of the original signal *x(t)* over time and frequency [49].

In the 2000s, the frequency domain has been highly employed with some significant results [48,51]. Indeed, Rantonen et al. [48] adopted the FFT to compute the FHRV in fetuses with cord arterial base deficit (8–12 mmol/L) and found a linear correlation between mid-frequency (0.07–0.13 Hz) spectral densities and the cord arterial base deficit values; this fact suggests that changes in the autonomic nervous cardiac control, reflected by a decreased FHRV, can occur in fetuses at risk of complications. Van Laar et al. [51] used the STFT and showed that fetal behavioral state and gestational age could cause considerable variability in the FHRV spectrum, with an increase in LF (0.04–0.15 Hz) and HF (0.4–1.5 Hz) power in near- and post-term fetuses during active sleep compared to quiet sleep. More recently, Cömert et al. [52] proposed a prognostic model for predicting fetal hypoxia, called image-based time-frequency (IBTF), based on the combination of STFT and grey level co-occurrence matrix (GLCM), where the STFT spectrograms are converted into 8-bit grayscale images whose characteristics (contrast, correlation, energy, and homogeneity) are used to classify FHR signals. The model was embedded in an open access software for CTG analysis [11] developed by the authors and showed promising accuracy (77.81%), sensitivity (76.83%), and specificity (78.27%) in recognizing hypoxic fetuses.

Still recently, the analysis of FHR in the frequency domain has been employed to study the evaluation of ANS and to investigate its development as a function of the gestational age. Obtained results, among others, showed that the LF/HF ratio in the normal pregnancy group slightly decreases over the gestational period [46]. This result is in accordance with the previous results of the authors [5].

#### 3.2.2. Autoregressive Models

A different method of FHR analysis can be found in AR models, which allow better identification of discrete frequency oscillations for time series and have been widely applied to the analysis of HRV and FHRV [24,46,49].

An AR series is expressed by the following equation:

(8) $$x\left(n\right)=-\overset{p}{\underset{k=1}{\sum }}a_{k}*x\left(n\right)+e\left(n\right)$$

where *x(n)* is the input signal, *p* is the model order, *a_k_* are the AR model parameters, and *e(n)* is the error, which is a zero-mean white Gaussian noise [49].

Despite their large use, the effective use of AR models mainly lies in the appropriate choice of the order of the model. Since the use of low-order AR models could prevent a comprehensive representation of the FHR signal properties, a high-order AR model could be preferred. Indeed, due to higher degrees of freedom, high-order AR models can better fit the time series.

Among the numerous criteria to estimate the appropriate model order, Akaike’s Information Criterion (AIC) [110] is one of the most used. It determines the order of the AR model as the one that minimizes the following function:

(9) $$\mathrm{AIC}\left(p\right)=N*\ln\;\sigma_{p}^{2}+2*p$$

where *N* is the number of data points and *σ_p_*^2^ is the mean squared error calculated as:

(10) $$\sigma^{2}=\frac{1}{N}\overset{N-1}{\underset{p=1}{\sum }}e^{2}\left(n\right)$$

In their study, Bracale et al. [26] analyzed 50 real CTG recordings through AR models with orders ranging from 4 to 14 and chose the best order using the AIC function and the whiteness test. Once the AR coefficients are known, they calculated the PSD of the FHRV as:

(11) $$S\left(f\right)=\frac{\sigma^{2}T}{{\left|1-\sum_{k=1}^{p}a_{k}e^{-j2\pi fkT}\right|}^{2}}$$

where *T* is the sampling period [26,49]. Their analysis confirmed eight as the optimum order to estimate the PSD of the FHRV for all signals under study.

While AR models provide better identification of discrete frequency oscillations for non-stationary and relatively short data records (30 s–60 s duration is usually an acceptable range), Fourier analysis, when applied to relatively stationary data records of more than 250 s, allows searching for very-low-frequency oscillations and performs better as an amplitude estimator when reporting the percent power in various frequency bands [6].

Already in 1989, Ansourian et al. [53] presented a digital analysis using AR power spectral estimation with an optimized Burg algorithm, which showed to provide good modeling of short-time series, potentially useful in the monitoring of fetal breathing movement, and a higher resolution than classical FFT, despite the increased computational complexity. Far later, in 2001, Cazares et al. [54] used an AR model of uterine activity (UA) to estimate the power at the contraction frequency. Results on 12 intrapartum UA traces were compared with the visual inspection of experts and confirmed that the proposed tenth-order AR model, with an agreement percentage of 62%, could be a helpful confidence index for the automated assessment of UA, potentially reducing errors made in the identification of uterine contractions. More recently, Fuentealba et al. [24] proposed a time-varying AR model to analyze the spectral dynamical changes in the FHR signal over time. They showed that certain time domain alterations could cause significant dynamical frequency domain changes and, in particular, that decelerations leave singular spectral traces, whose characteristics could help to distinguish between normal and pathological fetal conditions.

#### 3.2.3. Wavelet Transform

WT is used to analyze non-stationary signals or signals containing multiple components, thus being particularly useful for processing medical and biological signals [23].

In the case of FHR, it proved to be helpful in investigating the long-term non-stationary behavior of FHR [55].

The algorithm consists of the convolution of ‘local’ wavelike functions, namely, wavelets, with the signal under study, as reported in the following equation [23]:

(12) $$W_{s}\left(a,b\right)=\int_{-\infty }^{+\infty }s\left(t\right)\psi_{a,b}^{*}\left(t\right)dt$$

where *s(t)* is the time domain signal, * is the complex conjugate, and *ψa,b(t)* is the mother wavelet, scaled by a factor of *a* (*a* > 0) and dilated by the factor *b*, defined as:

(13) $$\psi_{a,b}\left(t\right)=\frac{1}{\sqrt{a}}\psi_{a,b}\left(\frac{t-b}{a}\right)$$

The appropriate selection of the type and width of the mother wavelet, i.e., by choosing one that matches the shape of the original signal at a specific scale and location, will give an effective transformation of the signal. However, this aspect can be critical in determining the performance of the analysis [111,112,113].

A WT is usually adopted to estimate the signal energy distribution through the calculation of the standard deviation of the wavelet coefficients in different frequency ranges of the FHR [55]. This allows the possibility of choosing the appropriate scale in the wavelet transform (smaller scales correspond to more rapid variations and therefore to higher frequencies) while ignoring the contribution of the other scales [56].

Wavelet transform has been employed in order to predict perinatal outcome [22] or to discriminate normal pregnancies from pregnancies complicated by gestational hypertension or gestational diabetes [23]. Both studies demonstrated that the use of parameters extracted from the wavelet domain contributes to revealing hidden information from FHR signals.

Vasios et al. [55] proposed an automated method to support the early diagnosis of fetal hypoxia using WT and self-organizing-map neural networks to analyze and classify FHR and fetal pulse oximetry signals. The application of WT allowed proper processing of the FHR signal, avoiding the problem of long-term non-stationary behavior as well as the extraction of the FHR power at different scale levels, which were used as input to the neural networks together with the pulse oximetry data. The classification performances showed good sensitivity (83%) and specificity (96%) in the categorization of the FHR patterns. The problem of fetal hypoxia was also addressed by Salamaleki et al. [56], who adopted the same system previously described by Vasios et al. [55] and confirmed that computerized analysis of FHR and pulse oximetry based on the combination of WT and ANN could give promising results in terms of both sensitivity (83.3%) and specificity (97.9%) for intrapartum diagnosis of hypoxic fetuses.

### 3.3. Nonlinear Techniques

Time and frequency analyses of FHR showed to be useful in the assessment of FHRV. However, significant limitations can be found in these so-called linear methods. On the one hand, time domain indices mainly rely on descriptive statistical measurements of the FHR signal, not being able to infer the physiological processes controlling the variation in the heart rhythm. On the other, frequency domain approaches showed to be sensitive to artifacts and do not allow the inspection of non-periodic trends embedded in the variability signal, leaving a shadow on the complex mechanisms underlying the variability of the FHR signal and thereby preventing a proper comprehension of the fetal health status and a reliable risk stratification. In addition, there is agreement on the fact that, similar to the adults’ HRV, the characterization of the FHRV through linear techniques is fundamentally limited in its power to describe the nonlinear structure of the underlying sympatho–vagal interactions [27,114,115].

The investigation of such hidden phenomena [12], whose understanding is fundamental to thoroughly describe the variability of biological signals such as the FHR, has been carried out using alternative methodologies, conventionally defined as ‘nonlinear techniques’, which include algorithms and approaches, applied alone or in combination with traditional time and frequency domain analyses, that aim to quantify non-periodic contributions to the variability of FHR signals and to classify them accordingly [17,57,58,59,116,117].

#### 3.3.1. Entropy Measurements

The quantification of regularity and irregularity or randomness in the heartbeat fluctuations can be achieved by using entropy measurements, which allow inferring the level of complexity in time series such as FHR. Among them, the most used for FHRV estimation are Approximate Entropy (ApEn), Sample Entropy (SampEn), and Multiscale Entropy (MSE).

Approximate entropy (ApEn) quantifies the independent statistical irregularities in the time series data [65,66,68,118], i.e., the ‘extent of randomness’ in sequences or time series [61]. Mathematically, the ApEn(*m,r*) measures ‘*the negative logarithmic likelihood that runs of patterns, which are close (within tolerance r) for k–1 observations, remain close on the next incremental comparison*’ [61].

In the case of FHR, this translates into the comparison of distances between *m*-dimensional *RR* patterns found in the *r*-neighborhood and the *RR* patterns obtained by increasing the number of vector components by one [61]. It is worth noting how, according to [119], the choice of those parameters for the calculation of the ApEn is a crucial factor.

The lower the ApEn, the higher the regularity and predictability of the signal [120], potentially allowing the distinction between a wide variety of systems: deterministic, stochastic, chaotic, etc. [63,118,121,122]. While the main advantage lies in the applicability to small and noisy datasets [118], the consistency of the taken measurements is highly dependent on the length of the time series [123]. This limitation can be overtaken by using SampEn, which is independent of the size of the dataset and adopts a simplified algorithm to detect similarities among signal trends [123,124]. Despite this, SampEn appears to be sensitive to predetermined parameters [125].

In general, the interpretation of entropy values can be difficult since variations in entropy can be due to signal artifacts (e.g., outliers) rather than changes in regularity [67,126].

The Approximate Entropy method ApEn was used in the analysis of FHR by Signorini et al. in 2003 [17,67]. The authors compared spectral parameters of Autoregressive models and nonlinear algorithms. The study was performed on 14 normal fetuses, 8 cases of gestational diabetes, and 13 fetuses with intrauterine growth retardation pregnancy. The analysis showed a significant difference between the groups under study, and the computed spectral parameters proved the ability to distinguish among normal and pathological subjects [17].

Lim Jongil used ApEn to assess fetal stress and lack of oxygen, as well as the metabolic and respiratory acidosis in women at the end of their pregnancy. The FHR of singleton fetuses >37 weeks with at least half an hour of records of CTG monitoring before delivery were analyzed. It was found that the indices ApEn and SampEn are significantly different in relation to the stage of labor during which these are measured [62].

ApEn and SampEn have been used to analyze the variability of the parameters in the time domain of 46 MCG fetal recorded in the range of 21–38 weeks by Moraes et al. [64]. They demonstrated the advantage of SampEn over ApEn in overcoming the limitations related to the length of the recordings and the lack of consistency in the quantification of the regularity of the signals.

The identification of antepartum fetal distress was performed by Magenes et al. in 2004 [60] through multiparametric analysis using approximate entropy (ApEn) and SVM, a method of supervised learning for pattern regression and classification. In the study performed on 70 cases (35 healthy and 35 pathological), the combined analysis through ApEn and SVM showed good accuracy (78%), specificity (78%), and sensitivity (79%) in distinguishing the two groups.

#### 3.3.2. Symbolic Dynamics Analysis

SD consists of transforming the inter-beat intervals time series into a series of ‘abstract’ symbols so that the signal’s samples are reduced to a few possible patterns of symbols, i.e., strings of consecutive symbols named ‘words’, then grouped in subgroups or ‘families’ [70].

An example of the methodology, as applied in the analysis of the HRV of adult subjects, is provided by Cysarz et al. [127], where, starting from a series of interbeat intervals *RR(i)* (*i = 1,…,* N), a binary symbolic series *S(i)* was created using the differences Δ*RR(i) = RR(i) − RR(i − 1)* between successive inter-beat intervals, and applying the following rule:

(14) $$S\left(i\right)=\left\{\begin{array}{c} 0\;\;\;if\;\Delta RR\left(i\right)\geq 0\\ 1\;\;\;if\;\Delta RR\left(i\right)<0 \end{array}\right.$$

Hence, decelerations of the instantaneous heart rate are symbolized by 0 s and accelerations by 1 s. Then, from the binary series *S(i)*, short binary sequences can be extracted simply by taking k consecutive symbols.

In this way, it is possible to simplify the study and classification of the dynamic of the system [128]. As for other nonlinear techniques, the use of this type of analysis could be helpful when used together with traditional methods in order to improve the reliability of the risk stratification. Indeed, the SD proved to be able to identify complex fluctuations in the heart rate better than traditional methods [70]. On the other hand, it should be taken into account that the choice of the alphabet of symbols and the criteria to form words of symbols can influence the obtained results and that the codification into symbols can cause loss of information and be influenced by the presence of outliers [129,130].

Van Leuween et al. [70] used SD and ApEn to categorize short segments of the FHR signals with respect to their degree of regularity and quantified the changes in regularity and irregularity over time. Their results proved an increase in the short-term complexity of the FHR over the gestational age due to the development of behavioral states, not only related to the HR but also to the motility, hemodynamics, metabolism, and response to stimulation. Such states and the transitions between them increase both irregular and regular HR patterns, which can be detected and properly interpreted using the proposed complexity measure.

In previous works [4,27,71,72], FHRV analysis through the SD was carried out, and this technique was compared with other traditional indices. Results showed how the SD is a more reliable measure than frequency domain parameters in assessing fetal development during the course of pregnancy, in accordance with the above-reported results. In addition, the ease of use and implementation of the methodology allows immediate interpretation of the CTG traces. Furthermore, in a recent work, it has been proved how SD analysis of healthy fetuses near to term is a powerful tool in classifying CTG traces according to their degree of variability [4] as well as to the type of delivery (spontaneous and cesarean) [27].

Since the patterns in FHR fluctuations change with gestation age, it can be assumed that maternal–fetal cardiac coupling may also evolve with maturation. Therefore, in their work, Khandoker et al. [69] investigated the nature of nonlinear interaction of maternal–fetal heart rate phase synchronization using a modified version of the SD, namely, high-resolution Joint Symbolic Dynamics (HRJSD). They estimated the degree of short-term cardiovascular coupling in growing fetuses. The HRJSD approach is characterized by three symbols, a threshold (individual dynamic physiological variability) for time series transformation, and 8 coupling pattern families (resulting in 64 different coupling patterns), which quantify patterns of the autonomic regulation. They concluded that HRJSD is a helpful tool for measuring the degree of fetal-maternal coupling, which could be a clinical marker of healthy prenatal development and fetal cardiac anomalies.

More recently, Montalvo-Jaramillo et al. [28] investigated the analytical value of SD in fetuses at term during low-risk labor, exploring the relationship between symbolic parameters (such as the percentage of no variations between three successive symbols, reflecting sympathetic modulations, and the probability of low variability with a threshold of 4 ms, usually associated with a low variability), and classic linear indices of FHRV, with particular attention to the PNN5, which is a time domain index representing the percentage of differences between successive normal (i.e., after removal of abnormal beats) inter-beat intervals exceeding 5 ms. The latter is defined as the percentage of differences between adjacent RR intervals larger than 5 ms, which is a time domain index employed to assess fetal development [73]. They confirmed that the SD could be a potential source of clinical biomarkers to differentiate the fetal autonomic cardiac condition at different stages of pregnancy.

#### 3.3.3. Fractal Analysis

Another method for the evaluation of FHRV is fractal analysis. The analysis of the fractal dimension allows calculating the degree of irregularity of a system by splitting it into a number of fundamental units (fractals) having the same shape at different scales of observation [14]. For those physiological signals which do not show a characteristic time scale, which is the case of a signal *y(t)* which has the same statistics (mean, variance, etc.) of its scaled version *y(at)* [131], the fractal analysis represents a measure of the invariance of the signal at different time scales.

In the case of FHR, the use of fractal analysis helped not only in the discrimination between pathological and normal FHR traces but also in the investigation of the nature of those long-range correlations in FHR fluctuations being manifested from around the twenty-fourth gestation week, which is a particularly relevant period from a physiological point of view since it coincides with major developments in the functional maturation of the ANS [14,76]. Nevertheless, it is worth highlighting that this analysis is more suited to time series of normal-to-normal interbeat intervals from long-term recording [132].

In their study, Di Rienzo et al. [74] used fractal analysis to identify pathological fetuses from 20 min noisy CTG traces. They performed the fractal analysis and were able to reliably differentiate between healthy fetuses and distressed ones. The potential of fractal features has also been proven by Felgueiras et al. [14], who compared them with traditional STV indices and demonstrated the better classification performance (error rate between 7% and 16%) of fractal dimensions over STV values in discriminating between pathological and normal FHR traces. Among different available algorithms to compute fractal features from FHR signals, Hopkins et al. [75] showed how most of the proposed fractal techniques offer significant improvement over simple standard deviation for the classification of compromised versus normal FHR traces. However, they also observed that no algorithm could completely separate all cases at all times, and that a clear separation can only be guaranteed in the longer term, making these features less suitable for real-time monitoring.

#### 3.3.4. Detrended Fluctuation Analysis

The DFA is a technique that helps in extracting nonlinear contributions in a signal. It is typically applied by segmenting the signal into short windows or blocks and by calculating two coefficients, *α*1 and *α*2, which measure the correlation between inter-beat intervals in the short and long term of a ‘detrended’ signal [133], i.e., after the elimination of continuous and linear components from the signal itself. The method has been widely exploited to carry out a correlation analysis in biomedical signals since it is able to remove external interference (noise) and thereby analyze only the intrinsic characteristics of the signal. Over other conventional fractal methods, DFA permits the detection of long-range correlations embedded in raw non-stationary time series [78,134] and quantification of self-similarities in time series [68].

However, the process of signal segmentation can produce two undesirable effects [135]: (i) if the signal length is not a multiple of the window length, at least one block will have fewer samples than the others unless we remove samples from the signal; (ii) when one block is considerably shorter than the others, then the energy of the signal within that block will be much lower than the rest of the signal, and discontinuities will be observed in the detrended signal. These two issues generally occur in the calculation of the α2 coefficient, which usually requires the use of large windows [77,136]. In addition, the DFA requires a high computational load, a minimum signal length of 8000 samples, and it is more suited to normal-to-normal interbeat intervals time series [130,134,137].

Despite the small number of studies on the applications of the DFA to the study of FHR, it is worth mentioning the work of Echeverrìa et al. [78], who evaluated the suitability of an enhanced DFA method for studying FHRV series and found that the enhanced DFA technique is able to highlight the presence of long-term fractality in FHRV, occurring at around 24 weeks of gestation, when well-known manifestations of fetal neural maturation arise.

#### 3.3.5. Poincaré Maps

Poincaré maps analysis is a graphical technique to obtain a quantitative and qualitative two-dimensional representation of the HRV and gather information about the heart’s behavior. A scatter plot is drawn by plotting the value of each inter-beat interval, *RR(n + 1)*, against the preceding one, *RR(n)*, and the obtained plot’s shape (namely, a cloud of points) can be then attributed to a specific pattern. Three indices can be calculated from the map to quantify the differences in the HRV [138]: (i) the standard deviation of the variability of RR intervals in the short term (minor axis of the cloud, SD1); (ii) the standard deviation of the variability of RR intervals in the long-term (major axis of the cloud, SD2); (iii) the axis ratio (SD1/SD2). The technique does not require continuous time series nor a normal distribution of the data. It provides an easily and immediately readable visual representation of the results and can be carried out also on a relatively short signal length (3–5 min is sufficient for FHRV analysis) [79].

A signal duration of 3–5 min is sufficient for FHRV analysis. However, the visual interpretation can lead to subjective evaluations [139,140], and the time-sequence information is lost in Poicaré plots, which only provide distributional information. Thus, the same plot can be generated by data sets with different underlying dynamics [141].

Poincaré maps are mostly employed to analyze HRV in adults, but some studies [25,79] also aimed at using Poincaré maps to determine the degree of chaos/uncertainty in the FHR variability and to evaluate dynamic changes in the FHR and dispersion of inter-beat intervals in healthy and pathological fetuses. Indeed, Mooney et al. [79] observed that the FHR pattern is chaotic and that this chaotic structure is maintained in short intervals of 3–5 min with dispersion in the variability that increases for shorter periods of observation.

**Figure 4:**
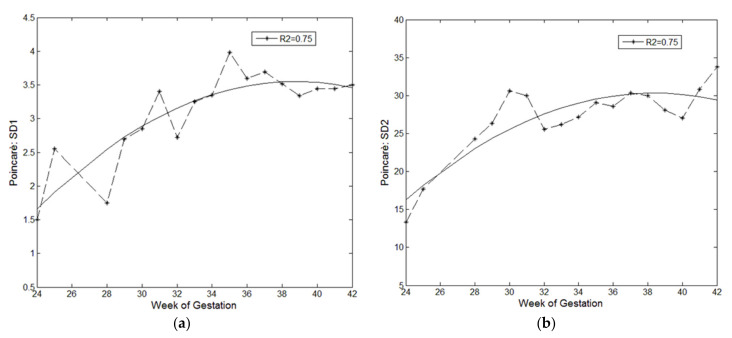
Trends of Poincaré indices according to the pregnancy period: (**a**) regression graphs of Poincaré SD1 parameter as a function of the gestational week; (**b**) regression graphs of Poincaré SD2 parameter as a function of the gestational week. For each graph, trend lines (dashed), fitting lines (solid), and R-squared values (R2) are reported.

For this study, we calculated the Poincaré SD1 and SD2 on a database of 187 FHR signals from real CTG traces of healthy fetuses between 24 and 42 weeks of gestation and found a correlation between both the computed Poincaré indices and the gestation period, as shown in Figure 4.

The determination coefficient (R2) of 0.75, obtained for both regression lines displayed in Figure 4, demonstrates how the Poincaré indices moderately reflect the growth and development of the fetus over the course of pregnancy.

#### 3.3.6. Hypothesis Tests Based on Surrogate Data

Before concluding the examination of nonlinear techniques, it is worth underling that they can sometimes lead to wrong conclusions about the behavior of the system due to heart rhythm fluctuations that are erroneously interpreted as caused by nonlinear dynamics. Therefore, in order to obtain more robust interpretations from the nonlinear analysis of the heart rate variations, the testing with surrogate data becomes an essential part of many of the above-mentioned methods, ensuring that the results are not achieved fortuitously, but they reflect actual properties and dynamics of the system under study. The tests with surrogate data allow determining if the output of the nonlinear analysis is related to deterministic nonlinear or stochastic linear properties of the signal [142]. Briefly, a surrogate is a dataset artificially generated by modifying certain features from the original signal. If the difference between the results obtained from the original signal and those obtained from the series surrogate is statistically significant, the hypothesis that the original series is generated by a stochastic process cannot be excluded [131]. The effectiveness of this method is highly dependent on the choice of the surrogates, which should be constructed preserving the properties of the original signal and should have the same spectral and cross-spectral characteristics as the original data. This can be obtained through randomization of the phases as follows:

- The two series (original and surrogate) are Fourier transformed;
- A random number uniformly distributed between 0 and 2π is generated and added to both phases of the Fourier transforms of the two series to preserve their difference (cross-spectrum);
- The two series are then anti-transformed.

The surrogate data test is commonly used in combination with other nonlinear techniques (fractal analysis, entropy measurements, etc.) [131,143].

#### 3.3.7. Overview of Advantages and Disadvantages of the Reviewed Techniques

The following table summarizes the main advantages and disadvantages of the major techniques of FHRV analysis explored in this review so far. It is worth underlining that, since the main results obtained by applying these methods to the analysis of the FHRV have been already described in the previous paragraphs, in Table 2, we focus more on the technical benefits and drawbacks of each method.

As we already mentioned, since most of the methodologies analyzed here have been firstly developed in studies on adult subjects and then applied to fetuses, most of the technical pros and cons reported in Table 2 are shared between FHRV and HRV.

As far as time and frequency domain parameters, the main drawback, widely recognized in both adults [111,112,115,144,145,146,147] and fetuses [4,5,17,27,114], consists of the scarce capability of investigating nonlinear physiologic dynamics of the system. A major limitation that is observed for both linear and nonlinear techniques is related to the lack of standardization and optimization (e.g., in the case of STV, AR models, WT, entropy indices, and SD). Another limitation that is shared between some linear and some nonlinear approaches is the dependency on signal characteristics, such as length (e.g., in the case of Fourier transform, entropy indices, DFA). Finally, some nonlinear methodologies are limited due to the complexity of the adopted algorithms (e.g., in the case of fractal analysis and DFA) or because they provide too synthetic indicators, thereby causing loss of relevant information (e.g., in the case of SD and Poincaré maps).

### 3.4. Other Methods for FHR Analysis

Other methods for processing and analyzing heart rate variations are also present in the literature, although their applications to the analysis of heart rate variations in fetuses are limited. Despite that, we believe that it is worth exploring the contributions brought by these additional techniques to the analysis of FHR signals, being aware that the obtained findings may need further validation and study in the context of FHRV.

#### 3.4.1. Hilbert and Hilbert–Huang Transform

The Hilbert transform (HT) consists of the convolution of a signal *x(t)* with the function 1*/(πt),* and it is expressed as:

$$H\left(t\right)=\frac{1}{\pi }\int_{-\infty }^{+\infty }\left(\frac{x\left(\tau \right)}{t-\tau }\right)d\tau$$

Despite HT being successfully applied several times to solve problems in the field of signal and image processing, only a few applications to the analysis of FHR are present in the literature [3,80,81,82,148].

Similarly, the Hilbert–Huang transform (HHT) was developed as a tool for the analysis of nonlinear and non-stationary data. The key of HHT is the empirical mode decomposition (EMD), consisting in the decomposition of a dataset into a finite small number of intrinsic mode functions (IMF), i.e., symmetrical signals with a unique local frequency (mono-component signal), which possesses a well-behaved Hilbert transform [149,150].

Compared with the traditional Fourier transform and WT, the EMD has no specified bases, meaning that its bases are adaptively produced depending on the signal to be decomposed. The obtained Hilbert spectrum of the generated IMFs provides a time–frequency distribution with sharp identification of the spectral components of the HR signal [149]. However, most of the studies using HT or HTT are limited to the analysis of HRV in adults.

In 2005, Ortiz et al. [151] applied EMD to extract HF (>0.3 Hz) components of FHRV data belonging to 10 fetuses at term with four different fetal activity conditions. They analyzed the effect of the fetal breathing or corporal movement on non-stationary FHR time series and observed that the mean HF power increases during abrupt and large accelerations or decelerations periods, regardless of the occurrence of fetal breathing and corporal movements. A multivariate extension of the EMD was proposed by Saleem et al. [30] to calculate robust features from FHR and uterine contraction signals in order to classify fetal states leading to ‘vaginal’ vs. ‘cesarean section’ delivery. The features extracted with this methodology were then used as input to machine learning algorithms, with the best classifier showing 91.8% sensitivity and 95.5% specificity. Recently, Marques et al. [3] proposed a system for computerized CTG based on HT. They combined HT with an adaptive threshold technique to detect fiducial points on CTG traces. The method showed good predictivity (around higher than 90%) for FHR accelerations and decelerations as well as uterine contractions.

#### 3.4.2. Lomb Method

With the Lomb method, an unevenly sampled signal is transformed, projecting the signal onto one element of an orthonormal base that minimizes the mean squared error energy *e(c_i_)*. The coefficient *c(i)*, representing the transformed signal *x(t)*, is that which minimizes the following function [83,152]:

$$e\left(c_{i}\right)=\int_{-\infty }^{+\infty }{\left(x\left(t\right)-c\left(i\right)b_{i}\left(t\right)\right)}^{2}dt$$

which is the squared differences between the signal *x(t)* and its projection onto an orthogonal basis *b_i_(t)*, defined as:

$$c\left(i\right)=\int_{-\infty }^{+\infty }x\left(t\right)b_{i}\left(t\right)dt$$

Using this method, it is not necessary to interpolate noisy or ectopic beat detection, thus avoiding the spectrum distortion produced with Fourier- or AR-based techniques [83,152]. Despite this, the methods have slower computational speed compared to FFT or other Fourier-based methods [153]. In the HRV analysis, the Lomb periodogram proved to be a more appropriate spectral estimation tool for unevenly sampled time series compared to traditional Fourier methods since no explicit data replacement is made (or model assumed), and the PSD is calculated from only the known values [154].

In the FHRV, the Lomb method confirmed its suitability to the analysis of unevenly sample data and demonstrated to be insensitive to missing points and useful in the PSD estimation from CTG signals, especially if applied in combination with additional techniques [83,84].

Romano et al. [85] and Cesarelli et al. [83] used the Lomb method to estimate the PSD of the FHRV and the SVB index on simulated FHR traces to show how an inappropriate storage rate of CTG data can lead to an erroneous estimation of some important clinical parameters, such as the SVB. They confirmed that the interpolation process and the use of different storage rates significantly affected the PSD estimation and demonstrated that the evaluation of SVB on evenly spaced FHR series provides overestimated values.

#### 3.4.3. Matching Pursuits

In order to extract additional FHR characteristics that previous spectral analyses cannot reveal, the MP method has been proposed. It is a modified version of the WT method, which creates time–frequency representations of the signal energy without cross-terms. The FHR signal is decomposed into waveforms selected from a dictionary of time-frequency atoms, which are the dilations, translations, and modulations of a single-window function, *g(t)*, defined as [86]:

(15) $$g_{\gamma }\left(t\right)=\frac{1}{\sqrt{s}}g\left(\frac{t-u}{s}\right)e^{i\eta t}$$

where *η* is the frequency modulation, *u* is the translation, *s* is the scaling parameter, and *γ* is the set of parameters.

The MP proved to overcome the limitations of both Fourier and WT transform. Indeed, while classical Fourier and WT transform are not particularly suitable to represent non-stationary signals and narrow-frequency band signals, respectively, MP showed great potential in identifying multiple periodicities in highly non-stationary signals such as FHR [13].

In their work, Akay et al. [13] showed the high sensitivity of the MP in detecting perturbations in the normal dynamic pattern of FHR signals, such as sinusoidal fluctuations of multiple frequencies or burst-type activities, and in estimating the complex regulatory systems involved in the FHR control.

Moreover, Salamalekis et al. used the MP method for the analysis of FHR signal power in the VLF and LF frequency ranges to detect fetal hypoxia during labor [86]. They estimated the power of the FHR signal in four frequency ranges: Very Low Frequency (VLF) range (<0.04 Hz), Low Low Frequency (LLF) (0.04–0.08 Hz), Low Frequency (LF) (0.08–0.15 Hz), and High Frequency (HF) (>0.15 Hz), and found that the LLF recognizes the cases with lower cord arterial pH (sensitivity 78.5%, specificity 52.3%), while the VLF recognizes the cases with lower pH (72.2% and 59%) and the cases of non-reassuring FHR (sensitivity 64.2%, specificity 53.1%). The sensitivity and specificity of the VLF parameter were 72.2% and 59%, respectively, in recognizing the cases with low pH and 64.2% and 53.1% in recognizing non-reassuring FHR.

#### 3.4.4. Lyapunov Exponents

The spectrum of Lyapunov exponents is one of the most useful tools for the study of the dynamics of nonlinear systems. Lyapunov exponents are estimates of the average speed of convergence or divergence of the exponential trajectory of a dynamic system near an attractor. Therefore, they are used to classify the asymptotic behavior of a dynamic system and to determine the stability of quasi-periodic and chaotic regimes as well as the equilibrium points and periodic solutions of a given vector. More generally, they can provide a qualitative and quantitative characterization of the dynamic behavior of a system. Lyapunov exponents can be measured for both continuous- and discrete-time systems, and the positivity of these indices reveal the presence of chaotic behavior in the system, i.e., small differences in the initial conditions can produce enormously different trajectories of the system. The numeric value of the Lyapunov exponent gives a precise indication of the time after which the system dynamics become unpredictable [29,87].

Despite the Lyapunov exponent being infrequently used in the analysis of FHRV, some interesting results are reported by Kikuchi et al. [88], who calculated the largest Lyapunov exponent from 119 FHR traces of healthy normal fetuses, and found no significant changes according to gestational age. On the other hand, compared to healthy fetuses, they observed significantly lower values in 69 FHR traces of IUGR (Intra Uterine Growth Restriction) fetuses (*p* < 0.001).

#### 3.4.5. Hidden Markov Models

Another nonlinear technique can be found in the Hidden Markov Models (HMMs), which have been applied to the analysis of electrocardiograms in recent decades. Basically, HMM is a statistical modeling technique that represents a complex system using a probability density function that varies according to the state of an underlying Markov chain. The term ‘hidden’ comes from the fact that the states of the model cannot be directly determined from observations [155].

Georgoulas et al. [89] used two HMMs together with traditional time domain and frequency domain analyses to extract a set of parameters for the automatic classification of FHR tracings belonging to hypoxic and normal fetuses. They compared HMMs characterized by a different number of hidden states and obtained the maximum overall classification rate of 83% for seven hidden states, with high classification rates both for normal (85%) and abnormal (81%) cases.

#### 3.4.6. Complexity of Lempel Ziv

The complexity of LZ is a method of symbolic sequence analysis that measures the complexity of finite length time series and has been employed for the classifications of heart rate patterns [156]. The input of the algorithm is constituted by a finite sequence of symbols (string) belonging to a previously defined alphabet. Sometimes, the input string contains some parts (sub-strings), which are repeated several times. The idea of the algorithm is that of exploiting these repetitions to obtain a compression of the signal. The analysis concerns the rate of occurrence of specific subsets of strings and the calculation of the LZ complexity, whose values are larger at the increasing complexity of the time series.

In their studies, Ferrario et al. [91] compared traditional parameters that are derived from the FHR signals with ApEn, SampEn, and LZ. They showed that the LZ was the only index providing significant differentiation between healthy fetuses and fetuses with intrauterine growth restriction (IUGR). Again, Ferrario et al. [90] computed LZ and the multiscale entropy to analyze CTG traces from normal (17), severe IUGR (23), and non-severe IUGR (19) fetuses between the twenty-seventh and thirty-fourth week of gestation, finding that the proposed indices are able to identify the actual IUGRs and separate them from the physiological fetuses (sensitivity = 77.8%; accuracy = 82.4%). The capability of LZ to distinguish between normal and IUGR fetuses has been confirmed by Magenes et al. [92] in further work.

Despite the above-mentioned results, to the best of our knowledge, the LZ seems to be applied only to support the discrimination of IUGR fetuses, and most of the studies are carried out from the same research groups.

#### 3.4.7. Principal Dynamic Models

The Principal Dynamic Models (PDM) method is another technique employed to identify and quantify nonlinear components of heart rate fluctuations. It describes the dynamics of a system as a hierarchy of nonlinear systems, and the signal is transformed using Volterra–Wiener kernels based on the expansion of Laguerre polynomials (*k*0, *k*1, *....*):

(16) $$y\left(n\right)=k_{0}+\overset{M-1}{\underset{m=0}{\sum }}k_{1}\left(m\right)x\left(n-m\right)+\overset{M-1}{\underset{m_{1}=0}{\sum }}\overset{M-1}{\underset{m_{2}=0}{\sum }}k_{2}\left(m_{1,\;}m_{2}\right)x\left(n-m_{1}\right)x\left(n-m_{2}\right)+\ldots$$

where *x(n)* is the input, *y(n)* is the output, and *M* is the memory of the system.

Though the PDM is applied to characterize nonlinear physiological systems, it is not suitable for analyzing the non-stationary RR series, as for traditional PSD calculation methods [149]. In addition, most studies using PDM are limited to the analysis of HRV in adults.

### 3.5. Artificial Neural Networks for the Classification of FHR Signals

Despite the wide range of techniques for the analysis of FHR traces and their extensive employing for the investigation of the FHRV behavior, and the fact that CTG is recognized as an important ante- and intrapartum monitoring tool to assess fetal wellbeing, there is no consensus, nor an established gold-standard, agreed by the scientific community for the interpretation of CTG traces, nor for the classification of FHR recordings [19]. This is one of the main reasons for the still-growing demand for the computer-aided study of FHRV and automated classification of FHR signals.

As an emerging trend, also in the healthcare sector, ANN proved its potential and showed interesting and promising results in the analysis of FHR, thanks to its powerful pattern recognition and classification capabilities.

Already between the 1990s and the 2000s, some researchers attempted the use of ANN to study FHR signals from CTG recordings. In 1994, Marques de Sa’ et al. [93] presented two multilayer perceptron ANN models for the estimation and classification of the FHR baseline. They obtained promising results in terms of accuracy, with 97.7% correctly classified cases in the training set and 90% correctly classified cases in the test set. Promising results, in the same year, were also obtained by John Liszka-Hackzell [94], who proposed two types of ANNs, namely, the back-propagation network and the self-organizing map network, to categorize different types of CTG traces according to the presence of accelerations/decelerations, baseline, beat-to-beat variability, presence of tachycardia/bradycardia. Subsequently, in 1995, other studies aimed at evaluating the potential of ANNs in the interpretation and of nonstress test results [95,96], showing encouraging achievements in the discrimination between normal and abnormal nonstress tests, with almost 60% agreement between the ANN model and human experts in the classification of abnormal FHR records [95].

In the early 2000s, Magenes et al. [97] showed that relatively simple ANN architectures, trained with a limited number of cases, can act as good nonlinear classifiers of fetal pathological states. Tests on 25 normal, 5 diabetes, and 6 IUGR cases gave an error of 5.5%. In the following years, other works explored the capability of different ANN models to discriminate between normal, suspicious, and pathological conditions from the pattern of CTG data [94,98,99], with some ANN models showing better performances over other classification techniques, such as discriminant analysis and decision tree, even in the prediction of fetal distress [100].

Later, between 2015 and 2018, further studies compared different ANN models among them and with other machine-learning-based approaches for the classification of CTG recordings. The best models showed accuracy, sensitivity, and specificity of at least 80% each [19], with ANN models having better performances than other algorithms. In particular, Yılmaz [101] focused on three different types of ANNs and achieved the best results with a generalized regression neural network. Cömert et al. [102] showed the better performance of ANNs over SVM, random forest, radial basis function network, and extreme learning machine, as also confirmed in a further study [103]. The same authors also showed that the combined use of both linear and nonlinear features as input parameters of the ANN model could increase the accuracy in the prediction of fetal distress [57].

More recently, ANNs have been compared with other data mining algorithms not only in terms of accuracy but also with regard to time consumption, demonstrating that an ANN model can be a good choice for classifying CTG traces since it can provide high accuracy at a reasonable computational time [104]. Furthermore, in 2019, Zhao et al. [20] used convolutional neural networks to predict fetal asphyxia, demonstrating the effectiveness of the proposed tool, which showed accuracy, sensitivity, and specificity up to 98.34%, 98.22%, and 94.87%, respectively.

## 4. Discussion

Despite the huge research efforts made in improving the analysis of FHRV through automatic computerized analysis of FHR signals, a gold standard has not been achieved yet. Instead, the availability of a plethora of methods for FHRV analysis, ranging from linear to nonlinear approaches, makes it even more difficult to make direct comparisons between the results obtained with different techniques and starting from different datasets and hypotheses.

On the contrary, the examined literature revealed the fundamental difficulties in the implementation of both traditional and nonlinear methods, mainly consisting of: (i) noisy nature of physiological signals; (ii) finite length of the data series; (iii) non-stationarity of the signals.

Thus, the overall take-home message would suggest combining both linear and nonlinear FHRV indices in order to reach a more comprehensive interpretation of the physiological phenomena driving the FHR fluctuations. Such a combined approach, using both nonlinear and traditional analytic methods, showed promising results in adult HRV as well as in fetuses [17,57,58,59,116,117]. This reflects what has already been shown in adult subjects by Voss et al. [130,157], who suggested that the combination of different HRV parameters from both time and frequency domains as well as from nonlinear dynamics can improve diagnostic precision in the analysis of HRV. Moreover, this statement is also in agreement with further studies on FHRV, which showed that the choice of the optimal linear/nonlinear parameter combination could improve the classification between healthy and IUGR fetuses [158] and, in general, could be more effective in classifying FHR patterns and assessing ANS maturation [64,159,160].

Indeed, the combination of linear and nonlinear techniques could be helpful not only in overcoming peculiar limitations of a single specific approach but also in investigating hidden dynamics and complex relationships between the FHR and the fetal ANS functioning, behavior, and development. However, such an integrated approach, based on the rational combination of the above-described methodologies, is still far from being standardized, and the various attempts made in this direction provide encouraging but limited and non-definitive results.

According to our opinion and the recent literature, a promising alternative approach is one involving methodologies from the AI field, more specifically referring to machine learning techniques, which currently appear to be the future of medical diagnostics; indeed, from their first introduction in the medical arena, they showed encouraging results in the analysis of FHRV. In particular, a support vector machine (SVM), alone or in combination with other linear and/or nonlinear techniques, was also used to predict the risk of metabolic acidosis [161], to evaluate the fetal health state [21], and to detect fetal hypoxia [52]. With the objective to differentiate vaginal vs. cesarean delivery and normal vs. pathological pregnancy, multiple machine algorithms were applied in combination [30,162,163]. Moreover, ANN proved to be useful for the automatic classification of FHR recordings [18,19,102] and for predicting fetal acidemia conditions [20].

Furthermore, despite this extensive use of AI algorithms in FHR analysis, the majority of the studies do not incorporate responses of the fetus to UCs [30], which could be an additional useful factor to assess, together with FHR, especially in the context of CTG monitoring.

The huge potential of AI in medicine and, above all, its recognized capability of detecting very complex input–output relationships could be seen not only as the future of the automatic FHR analysis but also as the possible union ring between linear and nonlinear FHRV indices, i.e., a tool to integrate and connect the information provided by multiple methodologies in order to give a better, more meaningful, and more comprehensive interpretation of the FHRV.

## 5. Conclusions

In this work, a review of linear and nonlinear methods for the analysis of FHRV has been carried out, providing an overview of the basic principles, the main pros and cons, and some of the relevant results achieved in the literature. The results of the examined studies revealed that none of the techniques available were demonstrated to be absolutely better than the others. Instead, a combination approach, using both traditional and nonlinear FHRV indices, is employed in many of the reviewed works, with promising but not yet definitive results. On the one hand, the application of different indices can bring more information, enhancing and improving the understanding of the complex dynamics involved in the control of the fetal heart rhythm; on the other hand, the proposed combined approaches lack integration and standardization. The latter creates a gap between the possibility of measuring several features of the FHR signal and the capability of obtaining a comprehensive, correct, and shared interpretation of the FHR dynamics, able to convert the collected data into information that could be useful for gynecologists and healthcare professionals in determining the health status of the fetus.

The frontiers paved by the use of AI in the diagnostics field could fill this gap by using traditional and nonlinear FHR features as an input to advanced machine learning algorithms that can improve the classification accuracy, enabling more reliable diagnostic information by distinguishing between healthy and pathological fetuses. This approach could increase the degree of integration between the most relevant analytic methods and, possibly, give an answer to the unmet clinical need of automatic analysis of FHR traces.

## Figures and Tables

**Table 1:** Most representative studies included in the qualitative synthesis of the review.

Approach	Technique	Acronym	Reference
Time domain measurements	General Description	-	[7,8,9,10,40]
	Short-Term Variability	STV	[9,22,25,41,42,43,44,45]
	Long-Term Variability	LTV	
	Interval Index	II	
	Long-Term Irregularity	LTI	
Frequency domain analysis	General Description	-	[5,6,17,46,47,48]
	Fast Fourier Transform	FFT	[5,11,38,46,48,49,50,51,52]
	Short Time Fourier Transform	STFT	
	Autoregressive Models	AR	[6,24,26,46,49,53,54]
	Wavelet Transform	WT	[22,23,55,56]
Nonlinear methods	General Description	-	[12,17,57,58,59]
	Entropy Indices	ApEn ^1^	[17,60,61,62,63,64,65,66,67,68]
		SampEn ^2^	
		MSE ^3^	
	Symbolic Dynamics	SD	[4,27,28,69,70,71,72,73]
	Fractal Analysis	-	[14,74,75,76]
	Detrended Fluctuation Analysis	DFA	[68,77,78]
	Poincaré Maps	SD1 ^4^	[25,79]
		SD2 ^5^	
Other methods	Hilbert–Huang Transform	HHT	[80,81,82]
	Lomb Method	-	[83,84,85]
	Matching Pursuit	MP	[13,86]
	Lyapunov Exponents	-	[87,88]
	Hidden Markov Models	HMM	[89]
	Lempel Ziv Complexity	LZ	[90,91,92]
Artificial Intelligence	Artificial Neural Networks	ANNs	[19,20,57,93,94,95,96,97,98,99,100,101,102,103,104]

^1^ Approximate Entropy. ^2^ Sample Entropy. ^3^ Multiscale Entropy. ^4^ Standard deviation 1, perpendicular to the line-of-identity (Poincaré maps). ^5^ Standard deviation 2, along the line-of-identity (Poincaré maps).

**Table 2:** Main pros and cons of the most used techniques for the analysis of FHRV.

Approach	Technique	Main Pros	Main Cons
Time domain measurements	General considerations	Despite their widespread use and recognized clinical value, time domain indices rely mainly on descriptive statistical measurements of the FHR and, therefore, they do not allow inferring the physiological processes controlling the variation in the heart rhythm.	
	STV	It correlates well with the development of metabolic academia, and it is recognized as a valuable antenatal monitoring tool.	There is no agreement on the formula used to calculate the STV, and its effectiveness can be influenced by frequency oscillations.
	LTV	It shows good sensitivity to sinusoidal fluctuations in the heart rhythms.	It can be complex to quantify numerically.
Frequency domain analysis	General considerations	Even though frequency domain indicators are widely employed and studied in the literature, particularly for their capability of investigating periodic trends in the heart rate fluctuations, it should be taken into account that these parameters are sensitive to artifacts and, since the heart is not a periodic oscillator, do not allow the inspection of non-periodic trends or transient changes embedded in the variability signal. In addition, power spectral indices are not able to characterize nonreciprocal changes of sympathetic and parasympathetic modulations.	
	Fourier Transform	It is relatively simple and does not require high computational power; therefore, it is widely employed in the literature. Specific conditions such as the cord arterial base deficit and changes in the behavioral state as well as in the gestational age cause variations in the FHRV spectrum.	The stationarity of the FHRV signal is an essential requirement.Limitations in describing the nonlinear structure of sympatho–vagal interactions. The length of data segments influences the frequency resolution.
	AR models	They provide better identification of discrete frequency oscillations for non-stationary and relatively short time series.	The determination of the optimal order of the AR model is not trivial, and a wrong choice can compromise the reliability of the model.
	WT	It allows proper processing of the FHR signal, avoiding the problem of long-term non-stationary behavior as well as the extraction of the FHR power at different scale levels. It uses short windows at high frequencies and long windows at low frequencies, thereby obtaining more precise spectral components and enabling a multi-resolution time-frequency representation of the signal.	There is still a lack of a gold standard procedure for the use of WT in the analysis of heart rate variations, even in the analysis of HRV in adults. In particular, it is not clear how the mother wavelet impacts the results and if results obtained using different mother wavelets can be compared. The performance can be unsatisfactory when more than one spectral component is present. Despite its better tunability compared to Fourier Transform, the time and frequency resolutions of wavelet transform cannot be arbitrarily good. Despite the undoubted theory advantages of the WT over traditional time-domain and frequency-domain analysis methods, there seem to be no direct comparisons with nonlinear techniques of FHRV analysis.
Nonlinear methods	Entropy indices (general considerations)	Entropy measurements provide a global index of the overall regularity of the time series under study, but they are not able to detect the dynamics that generate such behavior. In particular, entropy values should be interpreted carefully since they are not always a result of differences in regularity or complexity of the time series, but they can be an effect of the presence of outliers that affect the variance in the heart rate signal.	
	ApEn	It allows inferring the level of complexity of the FHR signal.	Results are highly dependent on the signal length and lack relative consistency since the algorithm also counts self-matches, thus introducing a bias in the results. The choice of the optimal parameters for the calculation of the ApEn is difficult.
	SampEn	Similar to ApEn, it allows quantifying the complexity of a time series, but it eliminates self-matches, requires lower computational time, and it is largely independent of the signal length.	The choice of the parameters to calculate the SampEn is critical, and there are no guidelines nor a gold standard on their use and optimization. It appears to be more sensitive than ApEn to noise and non-normal beats.
	SD	The signal’s samples are reduced to a few possible patterns of symbols, thereby simplifying the study and the classification of the underlying dynamics of the system.	The choice of the alphabet of symbols and the criteria to form words of symbols is complex, and a standard has not been achieved yet. The symbolization can cause loss of information and can be influenced by the presence of outliers.
	Fractal analysis	It allows calculating the degree of irregularity of the system by splitting it into a number of fundamental units (fractals) with the same shape at different scales of observation.	Algorithms for a reliable application of fractal analysis are not optimized for real-time monitoring. It is preferred for the analysis of time series of normal-to-normal interbeat intervals from long-term recording.
	DFA	Useful for removing external interference (signal noise). Over other conventional fractal methods, it permits the detection of long-range correlations embedded in raw non-stationary time series.	The signal segmentation can produce two undesirable effects: (i) if the signal length is not a multiple of the window length, at least one block will have fewer samples than the others; (ii) when one block is considerably shorter than the others, discontinuities will be observed in the detrended signal. A minimum signal length of 8000 samples and normal-to-normal interbeat intervals are requirements to apply the DFA. High computational load prevents the application of long series of data.
	Poincaré maps	They provide an easily and immediately readable visual representation of the results. A signal duration of 3–5 min is sufficient for FHRV analysis.	Visual interpretation can lead to subjective evaluation. SD1 and SD2 indices do not provide much additional information compared to time-domain measurements. Temporal information, which is crucial for the detection of nonlinear dynamics, is lost in traditional Poincaré analyses that focus on mere statistical indices extracted from a cumulative distribution of points.

## Data Availability

Not applicable.

## Abbreviations

AI: Artificial Intelligence; AIC: Akaike’s Information Criterion; ANN: Artificial Neural Network; ANS: Autonomous Nervous System; ApEn: Approximate Entropy; AR: Autoregressive; BPM: Beats Per Minute; CTG: Cardiotocography; DFA: Detranded Fluctuation Analysis; DFT: Discrete Fourier Transform; EFM: Electronic Fetal Monitoring; EMD: Empirical Model Decomposition; fECG: Fetal Electrocardiography; FFT: Fast Fourier Transform; FHR: Fetal Heart Rate; FHRV: Fetal Heart Rate Variability; fMCG: Fetal Magnetocardiography; fPCG: Fetal Phonocardiography; GLCM: Grey Level Co-occurrence Matrix; HF: High Frequency; HHT: Hilbert–Huang Transform; HMM: Hidden Markov Model; HRJSD: High-Resolution Joint Symbolic Dynamics; HRV: Heart Rate Variability; HT: Hilbert Transform; IBTF: Image-Based Time-Frequency; II: Interval Index; IMF: Intrinsic Mode Functions; IUGR: Intra-Uterine Growth Restriction; LF: Low Frequency; LTI: Long-Term Irregularity; LTV: Long-Term Variability; LZ: Lempel Ziv; MF: Middle Frequency; MP: Matching Pursuit; MSE: Multiscale Entropy; PDM: Principal Dynamic Models; PNN5: percentage of differences between successive normal inter-beat intervals exceeding 5 ms; PSA: Power Spectral Analysis; PSD: Power Spectral Density; SampEn: Sample Entropy; SD: Symbolic Dynamics SD1: standard deviation 1 perpendicular to the line-of-identity (Poincaré maps); SD2: standard deviation 2 along the line-of-identity (Poincaré maps); STFT: Short-Time Fourier Transform; STV: Short-Term Variability; SVB: sympatho–vagal balance; SVM: Support Vector Machine; UA: Uterine Activity; UC: Uterine Contraction; VLF: Very Low Frequency; WT: Wavelet Transform.
